# Mechanism of Exogenous Dopamine Regulating Shine Muscat Grape in Response to Low-Temperature Stress

**DOI:** 10.3390/plants14203225

**Published:** 2025-10-20

**Authors:** Jiaxin Li, Qiujie Wu, Jiahui Cheng, Jingxuan Zhu, Peisen Su, Jiayuan Wu, Xiucai Fan, Guirong Li

**Affiliations:** 1 School of Horticulture and Landscape Architecture, Henan Institute of Science and Technology, Xinxiang 453003, China; dazjydb@163.com (J.L.); qiushougrape@163.com (Q.W.); jiahuic0808@163.com (J.C.); xuangrape@163.com (J.Z.); 15565991852@163.com (P.S.); 15290398805@163.com (J.W.); 2 Zhengzhou Fruit Research Institute of Henan Province, Chinese Academy of Agricultural Sciences, Zhengzhou 450009, China; fanxiucai@caas.cn

**Keywords:** Shine Muscat grape, low-temperature stress, dopamine, physiological mechanism, gene expression

## Abstract

To reveal the mechanism by which exogenous dopamine (Da) regulates Shine Muscat grape (*Vitis labrusca* L. × *Vitis vinifera* L.) in response to low-temperature stress, annual Shine Muscat grape plants were used as material. Different concentrations of Da (0.2–1.0 mmol L^−1^) were set to investigate its synergistic regulatory effects on grape photosynthetic protection, osmotic adjustment, ion homeostasis, antioxidant defense, and cold-responsive gene expression and to identify the optimal concentration and core pathways through correlation analysis. The results showed that low-temperature stress significantly inhibited plant growth, reduced photosynthetic efficiency, disrupted ion balance, induced oxidative damage, and downregulated the expression of cold-responsive genes. Da exhibited a “low-concentration promotion and high-concentration inhibition” effect, with the 0.4 mmol L^−1^ treatment showing the best performance: growth indicators such as plant height and stem diameter increased by 22.4–52.2% compared with the low-temperature stress group; photosynthetic parameters and photosystem II (PSII) function were significantly improved; proline content increased by 40.3%; the Na^+^/K^+^ ratio decreased by 44.8%; activity of antioxidant enzymes such as superoxide dismutase (SOD) and peroxidase (POD) increased by 31.7–49.5%; and the expression of genes in the C-repeat binding factor (*CBF*) family was upregulated. Correlation analysis confirmed that the activity of SOD and catalase (CAT) showed a highly significant positive correlation with growth indicators (r > 0.8, *p* < 0.01) and a highly significant negative correlation with malondialdehyde (MDA) content (r < −0.8, *p* < 0.01), indicating that antioxidant defense is the core pathway. In conclusion, exogenous Da enhances the cold tolerance of Shine Muscat grape through multi-pathway synergy, with 0.4 mmol L^−1^ the optimal concentration, which can provide a theoretical basis for cold-resistant cultivation of grapes.

## 1. Introduction

Grape (*Vitis* spp.) is a perennial vine fruit tree that is widely planted throughout the world and plays an important role in the fields of fresh food, winemaking, and drying [1]. Shine Muscat grape (*Vitis labrusca* L. × *Vitis vinifera* L.) is favored in domestic and foreign markets due to its unique sweet taste, rich rose aroma, and good storage–transport resistance, creating substantial economic benefits for the grape industry [2]. However, under the context of global climate change, low-temperature stress has become a key environmental constraint that limits the growth, development, yield, and quality improvement of Shine Muscat grape [3]. Thus, exploring the regulatory mechanism of dopamine (Da) on its cold tolerance and proposing effective improvement strategies are of great practical significance.

Low-temperature stress causes multilevel harm to grapes: at the growth level, it inhibits germination, flowering, and fruit development, leading to reduced fruit set rate and deteriorated quality [4]; at the physiological level, it destroys the synthesis of photosynthetic pigments and the function of photosystem II (PSII), thereby lowering photosynthetic efficiency [5]; and at the cellular level, it increases cell membrane permeability, disrupts ion balance, and induces excessive accumulation of reactive oxygen species (ROS), ultimately resulting in membrane lipid peroxidation and metabolic imbalance [6]. In recent years, using exogenous substances to regulate plant stress resistance has become a research focus. A widely existing bioactive substance, Da has been proven to alleviate low-temperature stress damage in multiple crops by regulating proline content, antioxidant enzyme activity, and polyamine metabolism [7,8,9].

Despite the verified stress-resistant effect of Da in various crops [10,11], research on grapes—especially the Shine Muscat cultivar—remains insufficient. Dai et al. only reported the effect of Da on the photosynthetic performance and quality of Shine Muscat grape in the Hexi Corridor of Gansu Province without involving the regulatory mechanism of cold tolerance [12]. Existing studies on Shine Muscat cold resistance mainly focus on rootstock screening [13,14], traditional cold prevention measures [4], or other exogenous substances [5,11,12], while the specific mechanism of Da regulating Shine Muscat response to low-temperature stress—including its synergistic role in photosynthetic system protection, ion homeostasis maintenance, antioxidant defense, and cold-responsive gene expression—remains unclear. Additionally, previous studies on the physiological response of Shine Muscat saplings grafted with different rootstocks under combined salt and low-temperature stress indicated that ion homeostasis is the core of stress resistance, but whether exogenous Da can directly regulate this process still needs exploration [13]. Moreover, studies on the mechanism of exogenous substances improving grape cold tolerance point out that most regulatory substances have concentration-dependent threshold effects, yet the optimal concentration of Da for Shine Muscat and its multi-pathway synergistic mechanism under low-temperature stress are still unclear [15].

To address the current gap in research on the regulatory mechanism of Da in the cold resistance of Shine Muscat grapes, this study used 1-year-old Shine Muscat grape seedlings as experimental material, set up five gradient Da concentration treatment groups, and explored the multi-pathway synergistic regulatory network of Da in regulating the cold resistance of Shine Muscat grapes. This study aimed to verify the concentration effect law of Da on the cold resistance of Shine Muscat grapes and clarify the optimal Da concentration threshold for cold-resistance regulation. We analyzed the multidimensional synergistic mechanisms of Da in protecting the photosynthetic system, maintaining cellular osmotic balance, preserving ion homeostasis, activating the antioxidant defense system, and regulating cold-responsive gene expression in grape plants under low-temperature stress. The results of this study will help fill the theoretical gap in Da research related to the cold resistance of Shine Muscat grapes, provide directly applicable and precise technical parameters for the application of Da in cold-resistant cultivation practice of grapes, and further offer important theoretical support and practical reference for stress resistance genetic improvement and cultivation technology optimization of cold-sensitive fruit crops, thereby contributing to promoting technological breakthroughs and industrial application upgrading in the field of fruit tree cold-resistance research.

## 2. Results

### 2.1. Effect of Exogenous Dopamine on the Growth of Shine Muscat Grapes Under Low-Temperature Stress

**Figure 1:**
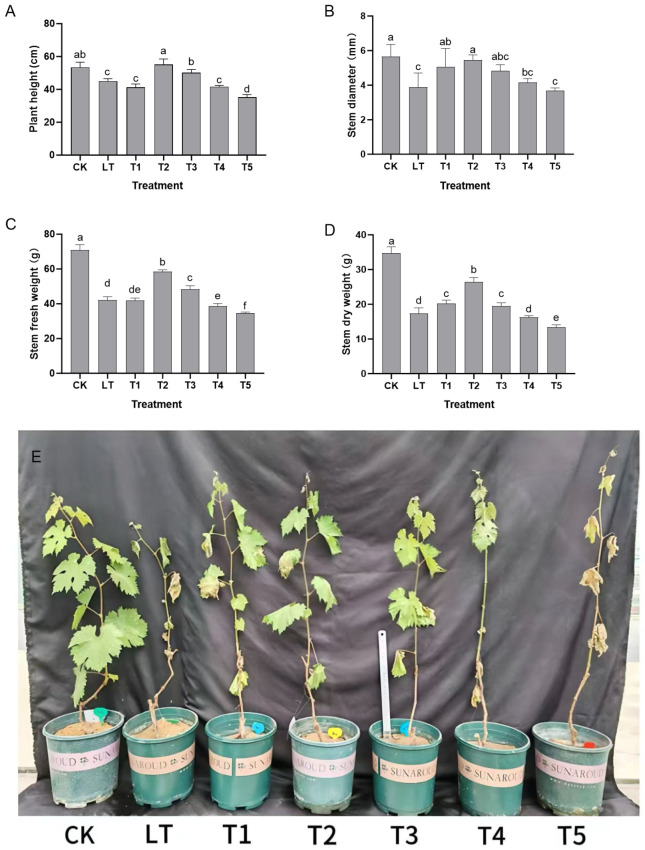
The impact of exogenous dopamine on the growth of Shine Muscat (*Vitis labrusca* L. × *Vitis vinifera* L.) grapes under low-temperature stress. (**A**) Plant height; (**B**) stem diameter; (**C**) fresh weight of stem; (**D**) dry weight of stem; (**E**) morphological differences of Shine Muscat grape seedlings under different treatments. Abbreviations: CK = control group (normal temperature: 25–28 °C, 1/2 Hoagland nutrient solution irrigation); LT = low-temperature stress group (0 °C, 1/2 Hoagland nutrient solution irrigation); T1–T5 = dopamine treatment groups under low-temperature stress with dopamine concentrations of 0.2, 0.4, 0.6, 0.8, and 1.0 mmol L^−1^. Replication information: Each treatment included 8 biological replicates (seedlings) and 3 technical replicates per seedling; data represent the average of 8 seedlings × 3 technical replicates. Statistical note: Different lowercase letters indicate significant differences among treatments at the *p* < 0.05 level (one-way ANOVA, Duncan’s multiple comparison).

Under normal growth conditions (CK group), the plant height reached 53.4 cm, the stem diameter 5.65 mm, the fresh stem weight was 70.88 g, and the dry stem weight was 34.75 g. The overall growth was characterized by upright and straight new shoots, a robust and resilient stem base, fully spread and lustrous leaves, and a coordinated development of nutrient accumulation, cell elongation, and substance synthesis (Figure 1).

Low-temperature stress (LT group) significantly disrupted this growth balance. The appearance of the seedlings showed a significant decline: the plant height dropped to 45.03 cm (a decrease of 15.7%), the stem diameter shrank to 3.89 mm (a reduction of 31.1%), and the fresh stem weight and the dry stem weight decreased to 42.13 g (a reduction of 40.6%) and 17.34 g (a reduction of 50.1%), respectively (Figure 1A–D). The plant became shorter overall, the stem became thin and weak, prone to lodging, the leaf edges curled slightly, and the leaf color became pale and lacked luster (Figure 1E). This directly demonstrated the overall inhibition of low temperature on the longitudinal elongation, lateral thickening, and substance accumulation of the plants.

After treatment with different concentrations of dopamine, the appearance of the seedlings showed a regular change pattern of “low promotion and high inhibition” with concentration: the 0.4 mmol L^−1^ treatment (T2 group) had the best effect, with a plant height of 55.13 cm, a stem diameter of 5.46 mm, a fresh stem weight of 58.48 g, and a dry stem weight of 26.39 g. Compared with the LT group, these values increased by 22.4%, 40.6%, 38.8%, and 52.2%, respectively. At this time, the new shoots of the seedlings regained their uprightness, the stems became as thick as those of the CK group, the degree of leaf spread increased, and the leaf color deepened. The plant height and stem diameter even exceeded those of the CK group and all indicators were synergistically enhanced, highlighting the effective alleviation of low-temperature stress. When the concentration was increased to 0.6 mmol L^−1^ (T3 group), the appearance of the seedlings began to decline, the stem was slightly thinner than that of the T2 group, and the degree of leaf spread decreased. For ≥0.8 mmol L^−1^ treatment (T4 and T5 groups), the growth inhibition intensified: the plant height of the T5 group (1.0 mmol L^−1^) was only 35.17 cm, the stem diameter was 3.69 mm, the fresh stem weight was 34.58 g, and the dry stem weight was 13.43 g (lower than the LT group). Overall, the plants became significantly shorter, the stems became thin, fragile, and prone to lodging, and the leaves showed mild wilting and a pale color, reflecting the comprehensive inhibition of growth by high concentrations of dopamine (Figure 1).

In summary, the dynamic appearance of the seedlings in all six groups was highly synchronous with such growth indicators as plant height and stem diameter. This jointly confirmed the blocked growth state of Shine Muscat grapes under low-temperature stress and the concentration-dependent regulatory effect of exogenous dopamine. The 0.4 mmol L^−1^ treatment most effectively coordinate the longitudinal growth, lateral development, and substance accumulation of the plants and achieved the best alleviation of low-temperature stress by improving the plant type and growth condition.

### 2.2. Effects of Exogenous Dopamine on the Protection and Efficiency Pathways of the Photosynthetic System of Shine Muscat Grapes Under Low-Temperature Stress

**Figure 2:**
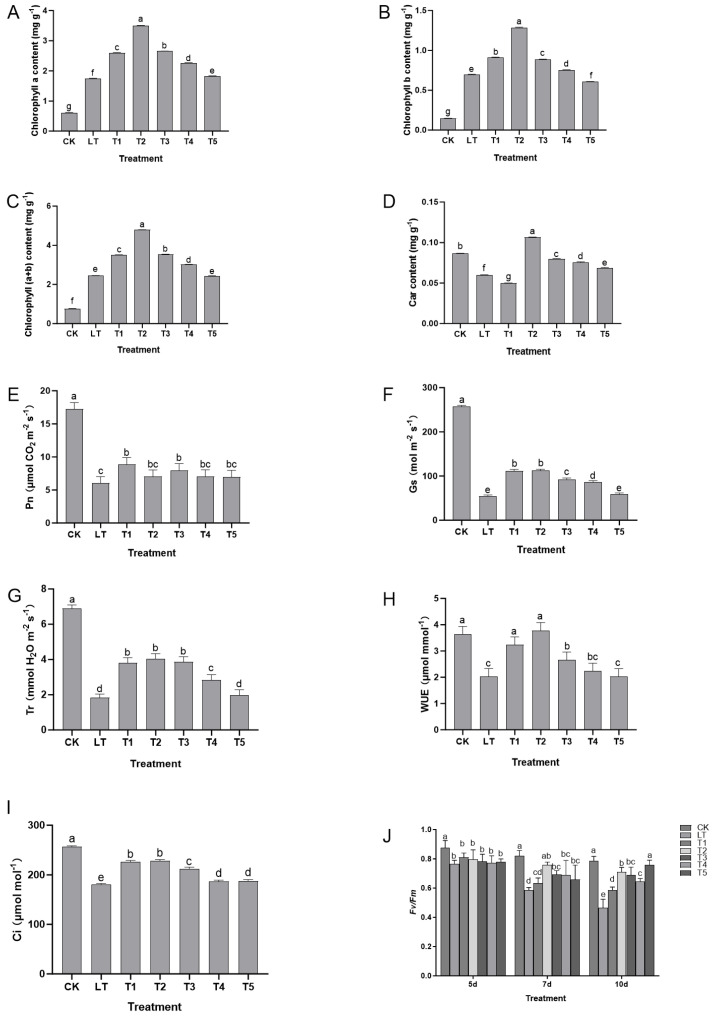
The impact of exogenous dopamine on the protection and efficiency pathways of the photosynthetic system in Shine Muscat grapes under low-temperature stress. (**A**) Chlorophyll a (Chla) content; (**B**) chlorophyll b (Chlb) content; (**C**) total chlorophyll (Chla + b) content; (**D**) carotenoid (Car) content; (**E**) net photosynthesis rate (Pn); (**F**) stomatal conductance (Gs); (**G**) transpiration rate (Tr); (**H**) water use efficiency (WUE); (**I**) intercellular CO_2_ concentration (Ci); (**J**) (Fv/Fm: maximum photochemical efficiency, ΦPSII: actual photochemical efficiency, ETR: electron transfer rate, NPQ: non-photochemical quenching. Data represent dynamic changes on days 5, 7, and 10 after treatment; each time point included 5 biological replicates and 3 technical replicates per seedling. Abbreviations: CK = control group (normal temperature: 25–28 °C, 1/2 Hoagland nutrient solution irrigation); LT = low-temperature stress group (0 °C, 1/2 Hoagland nutrient solution irrigation); T1–T5 = dopamine treatment groups under low-temperature stress with dopamine concentrations of 0.2, 0.4, 0.6, 0.8, and 1.0 mmol L^−1^. (**A**–**I**) Photosynthetic pigments/gas exchange: 6 biological replicates per treatment, 2 technical replicates per sample. (**J**) Fluorescence parameters: 5 biological replicates per treatment, 2 technical replicates per leaf. Statistical note: Different lowercase letters indicate significant differences among treatments at the *p* < 0.05 level.

Low-temperature stress significantly disrupted the structure and function of the photosynthetic system of Shine Muscat grapes. However, exogenous dopamine treatment alleviated the damage caused by low temperature and maintained photosynthetic efficiency by regulating key photosynthetic indicators (Figure 2). Under low-temperature stress, the content of chlorophyll (Chla, Chlb, and total Chl) in grape leaves significantly decreased (Figure 2A–C), which might have been related to the enhanced activity of chlorophyll-degrading enzymes or the inhibition of synthesis under low temperature, directly weakening the material basis for light absorption and conversion. The content of carotenoids (Car) also decreased simultaneously (Figure 2D), further affecting the light protection ability. The changes in photosynthetic gas exchange parameters corresponded to this: under low-temperature stress, the net photosynthesis rate (Pn), stomatal conductance (Gs), transpiration rate (Tr), and water use efficiency (WUE) significantly decreased, while the intercellular CO_2_ concentration (Ci) showed an upward trend (Figure 2E–I), indicating that the decrease in photosynthesis rate under low temperature was not solely caused by stomatal limitation (Gs decrease leading to insufficient CO_2_ supply), but might have been related to non-stomatal limitations (such as PSII function impairment, inhibition of dark reaction enzyme activity). At the same time, the function of PSII was severely damaged, manifested as significant decreases in the maximum photochemical efficiency (Fv/Fm), actual photochemical efficiency (ΦPSII), and electron transfer rate (ETR) and an abnormal increase in non-photochemical quenching (NPQ) (Figure 2J), This indicated the destruction of the PSII reaction center structure, a decline in light energy conversion efficiency, and an inability of excess light energy to be effectively consumed through the photochemical pathway, forcing it to be released as heat and exacerbating the potential damage to the photosynthetic system.

After exogenous dopamine treatment, the above indicators were significantly improved: the decrease in the content of chlorophyll (Chla, Chlb, and total Chl) and carotenoids (Car) was significantly inhibited (Figure 2A–D), maintaining strong light capture and light protection ability. At the same time, the decline in Pn, Gs, Tr, and WUE was significantly alleviated and the increase in Ci was reduced (Figure 2E–I), indicating that dopamine improved the photosynthetic system under low-temperature stress by optimizing stomatal behavior and alleviating non-stomatal limitations (such as stable PSII function), enhancing carbon assimilation efficiency. The decreases in Fv/Fm, ΦPSII, and ETR of PSII were reduced and the increase in NPQ was inhibited (Figure 2J), suggesting that dopamine can stabilize the PSII reaction center structure, reduce light energy conversion obstacles and excessive light energy heat dissipation, and alleviate light damage to the photosynthetic system.

In summary, exogenous dopamine alleviated the damage of low temperature to the photosynthetic system of Shine Muscat grapes through a multi-pathway synergy of pigment synthesis maintenance—stomatal regulation optimization—photosystem protection: at an appropriate concentration (0.4 mmol L^−1^), it maintained the content of chlorophyll and carotenoids to ensure light absorption and light protection, stabilized the function of PSII to promote light energy conversion, and coordinated stomatal and non-stomatal processes to improve carbon assimilation efficiency, thereby achieving the protection of the photosynthetic system under low-temperature stress and increasing its photosynthetic efficiency.

### 2.3. Effects of Exogenous Dopamine on Penetrative Regulation and Ion Homeostasis Maintenance Pathways of Shine Muscat Grapes Under Low-Temperature Stress

Low-temperature stress (LT group) significantly induced the accumulation of free proline in the leaves of Shine Muscat grapes (from 102.33 μg g^−1^ in the CK group to 353.23 μg g^−1^) while causing ion homeostasis imbalance, manifested as an increase in Na^+^ content from 1.01 mg g^−1^ to 5.37 mg g^−1^, and K^+^ from 6.50 mg g^−1^ to 9.33 mg g^−1^, but the Na^+^/K^+^ ratio increased sharply from 0.16 to 0.58, indicating that low temperature responded to dehydration stress by promoting the synthesis of permeation-regulating substances, but disrupted the selectivity of cell membranes and caused ion distribution disorder.

**Figure 3:**
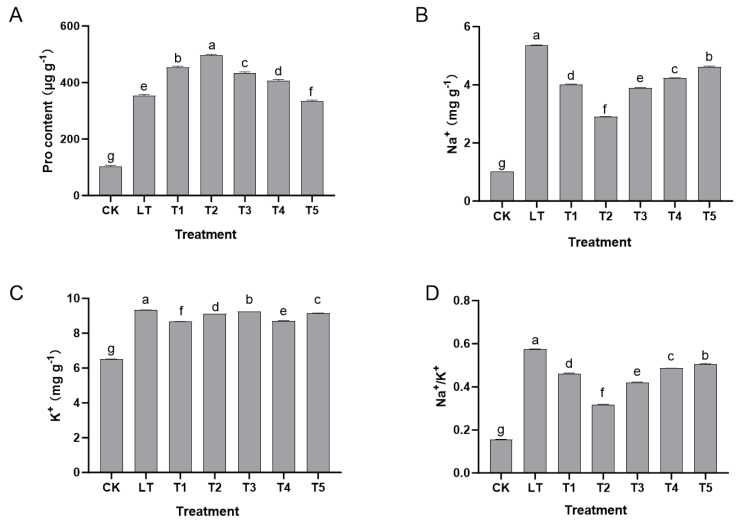
The impact of exogenous dopamine on the osmotic adjustment and ion homeostasis maintenance pathways in Shine Muscat grapes under low-temperature stress. (**A**) Proline (Pro) content; (**B**) sodium ion (Na^+^) content; (**C**) potassium ion (K^+^) content; (**D**) Na^+^/K^+^ ratio. For each treatment group, 6 seedlings were selected as biological replicates. From each seedling, 0.1 g of leaf tissue (mixed from 3 small leaf disks) was sampled for indicator determination, and all indicators were measured with 3 technical replicates. Abbreviations: CK = control group (normal temperature: 25–28 °C, 1/2 Hoagland nutrient solution irrigation); LT = low-temperature stress group (0 °C, 1/2 Hoagland nutrient solution irrigation); T1–T5 = dopamine treatment groups under low-temperature stress with dopamine concentrations of 0.2, 0.4, 0.6, 0.8, and 1.0 mmol L^−1^. Replication information: 6 biological replicates per treatment, 3 technical replicates per sample; ion content measured with 3 parallel injections per sample. Statistical note: Different lowercase letters indicate significant differences among treatments at the *p* < 0.05 level.

However, after exogenous dopamine treatment, these indicators significantly improved. Treatments of 0.2–0.6 mmol L^−1^ (T1–T3) enhanced permeation regulation and improved ion balance, with the 0.4 mmol L^−1^ (T2 group) treatment being the most effective: proline content reached 495.56 μg g^−1^ (an increase of 40.3% compared to the LT group), Na^+^ content decreased to 2.90 mg g^−1^ (a reduction of 45.9%), K^+^ remained at 9.12 mg g^−1^, and the Na^+^/K^+^ ratio decreased to 0.32 (a reduction of 44.8%). At this concentration, dopamine enhanced cell osmotic pressure by synergistically promoting proline accumulation, inhibited Na^+^ influx (or promote excretion), and stabilized K^+^ levels, achieving dual optimization of permeation regulation ability and ion gradient homeostasis. High concentrations (0.8–1.0 mmol L^−1^) of dopamine significantly reduced proline content (333.45–406.67 μg g^−1^), and their improvement effect on ion balance also weakened, indicating that excessive dopamine can weaken the coordinated regulatory effect of permeation regulation substance (proline) synthesis accumulation and ion (Na^+^, K^+^) transmembrane transport balance (Figure 3).

In summary, an appropriate concentration of exogenous dopamine (0.4 mmol L^−1^) optimized the Na^+^/K^+^ ratio by coordinating the synthesis accumulation of permeation-regulating substances (proline) and the transmembrane transport balance of ions (Na^+^, K^+^), thereby enhancing the adaptability of Shine Muscat grapes to low-temperature stress.

### 2.4. Effect of Exogenous Dopamine on the Antioxidant Defense Pathway of Shine Muscat Grapes Under Low-Temperature Stress

**Figure 4:**
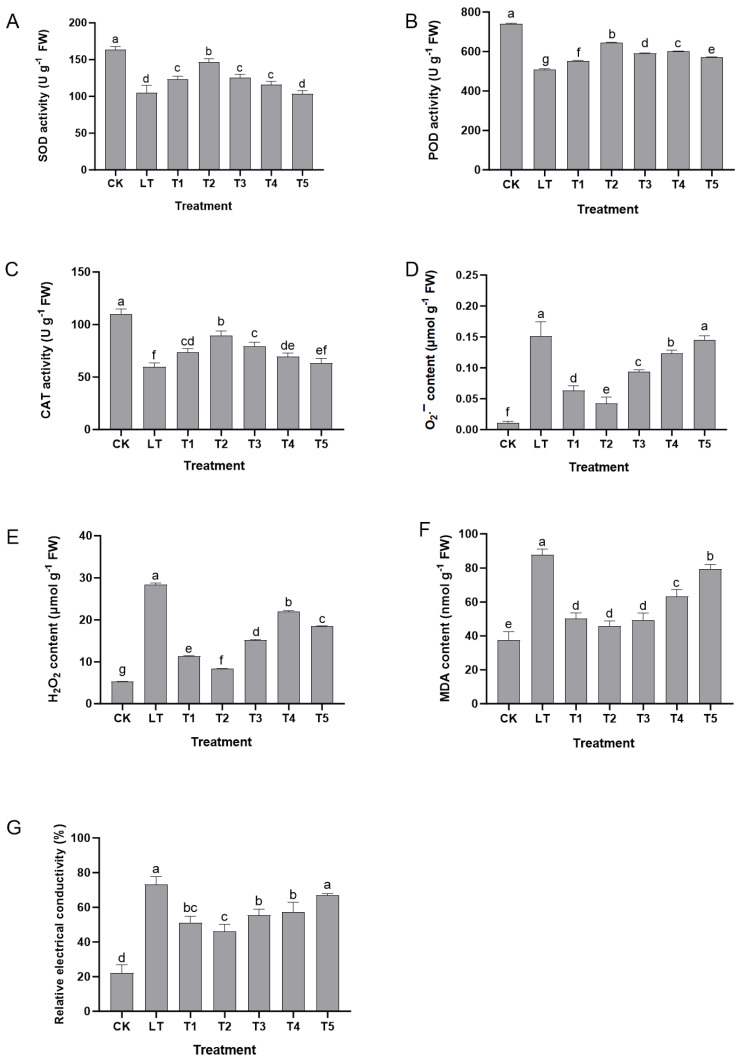
The effect of exogenous dopamine on antioxidant defense-related indicators in Shine Muscat grapes under low-temperature stress. (**A**) Superoxide dismutase (SOD) activity; (**B**) peroxidase (POD) activity; (**C**) catalase (CAT) activity; (**D**) superoxide anion (O_2_$\overset{-}{.}$) content; (**E**) hydrogen peroxide (H_2_O_2_) content; (**F**) malondialdehyde (MDA) content; (**G**) relative conductivity (REC). Each treatment included 6 biological replicates and 3 technical replicates per sample. SOD, POD, and CAT activity was measured with 3 parallel enzyme activity assays to ensure data stability. Abbreviations: CK = control group (normal temperature: 25–28 °C, 1/2 Hoagland nutrient solution irrigation); LT = low-temperature stress group (0 °C, 1/2 Hoagland nutrient solution irrigation); T1–T5 = dopamine treatment groups under low-temperature stress with dopamine concentrations of 0.2, 0.4, 0.6, 0.8, and 1.0 mmol L^−1^. Statistical note: Different lowercase letters indicate significant differences among treatments at the *p* < 0.05 level.

Low-temperature stress (LT group) significantly disrupted the oxidative balance of Shine Muscat grapes: compared with normal growth conditions (CK group), the activity of antioxidant enzymes such as superoxide dismutase (SOD), peroxidase (POD), and catalase (CAT) was significantly reduced, with SOD decreasing from 163.44 U g^−1^ to 111.43 U g^−1^, POD decreasing from 740.33 U g^−1^ to 509.55 U g^−1^, and CAT decreasing from 110.23 U g^−1^ to 59.77 U g^−1^, leading to enzymatic clearance of ROS. The ability had significantly weakened. At the same time, a large amount of oxidation products accumulated, with the content of superoxide anions increasing from 0.0112 μmol g^−1^ to 0.1511 μmol g^−1^, hydrogen peroxide (H_2_O_2_) increasing from 5.26 μmol g^−1^ to 28.39 μmol g^−1^, and membrane lipid peroxidation product malondialdehyde (MDA) increasing from 37.53 nmol g^−1^ to 87.77 nmol g^−1^. The relative conductivity (REC) also increased significantly, indicating that excessive accumulation of ROS under low-temperature stress had caused serious damage to cell membrane structure. The T2 group showed 31.7–49.5% higher SOD, POD, and CAT activity than the LT group, accompanied by 48.0–72.0% lower ROS and MDA content (Figure 4A–F). Correlation analysis confirmed that SOD and CAT activity was highly positively correlated with fresh stem weight (r = 0.979, 0.956; *p* < 0.01) and highly negatively correlated with MDA content (r = −0.941, −0.902; *p* < 0.01; Table 1), indicating that antioxidant enzyme activation is closely linked to growth recovery under low-temperature stress.

The regulation of oxidative imbalance by exogenous dopamine exhibited a significant concentration effect of “low promotion and high inhibition.” Treatment with 0.2–0.6 mmol L^−1^ dopamine (T1–T3 groups) enhanced antioxidant capacity to varying degrees, with the most significant effect observed in the 0.4 mmol L^−1^ (T2 group). At this concentration, the SOD activity reached 146.77 U g^−1^ (an increase of 31.7% compared to the LT group), the POD reached 643.98 U g^−1^ (an increase of 26.4%), and the CAT reached 89.36 U g^−1^ (an increase of 49.5%). The synergistic enhancement of enzyme activity significantly improved ROS clearance efficiency, reducing O_2_ content to 0.0423 μmol g^−1^ (a decrease of 72.0% compared to the LT group), H_2_O_2_ to 8.33 μmol g^−1^ (a decrease of 70.6%), and MDA to 45.67 nmol g^−1^ (decreased by 48.0%), and REC also decreased synchronously, indicating effective protection of cell membrane integrity. However, high concentrations of dopamine (0.8–1.0 mmol L^−1^, T4 and T5 groups) significantly weakened the antioxidant defense function, and the activity of SOD, POD, and CAT were significantly decreased compared to the T2 group (such as SOD in the T5 group being only 103.46 U g^−1^). At the same time, the content of O_2_$\overset{-}{.}$, H_2_O_2_, MDA, and REC was significantly increased (such as MDA in the T5 group rising to 79.44 nmol g^−1^), even approaching the level of the LT group, suggesting that excessive dopamine may inhibit the activation of the antioxidant system (Figure 4).

In summary, an appropriate concentration of exogenous dopamine (0.4 mmol L^−1^) can synergistically enhance the activity of antioxidant enzymes such as SOD, POD, and CAT, effectively eliminate excessive ROS induced by low temperature, and reduce membrane lipid peroxidation damage, thereby strengthening the antioxidant defense system of Shine Muscat grapes and alleviating low-temperature stress damage. However, high concentrations of dopamine had negative effects due to the inhibition of antioxidant function. These results provide important evidence for analyzing the physiological mechanisms of dopamine enhancing grape low-temperature tolerance.

### 2.5. Effect of Exogenous Dopamine on the Regulation Pathway of Cold-Resistance Gene Expression in Shine Muscat Grapes Under Low-Temperature Stress

Under normal growth conditions (CK group), all genes in the *CBF* family [16] maintained a certain basal expression level. Low-temperature stress (LT group) significantly inhibited the expression of these genes. Compared with the CK group, the relative expression levels of *CBF1*, *CBF2*, *CBF9*, *CBF11*, and *CBF15* significantly decreased (*p* < 0.05), indicating that the transcriptional activation ability of cold-resistant genes in Shine Muscat grapes was inhibited under low-temperature stress.

**Figure 5:**
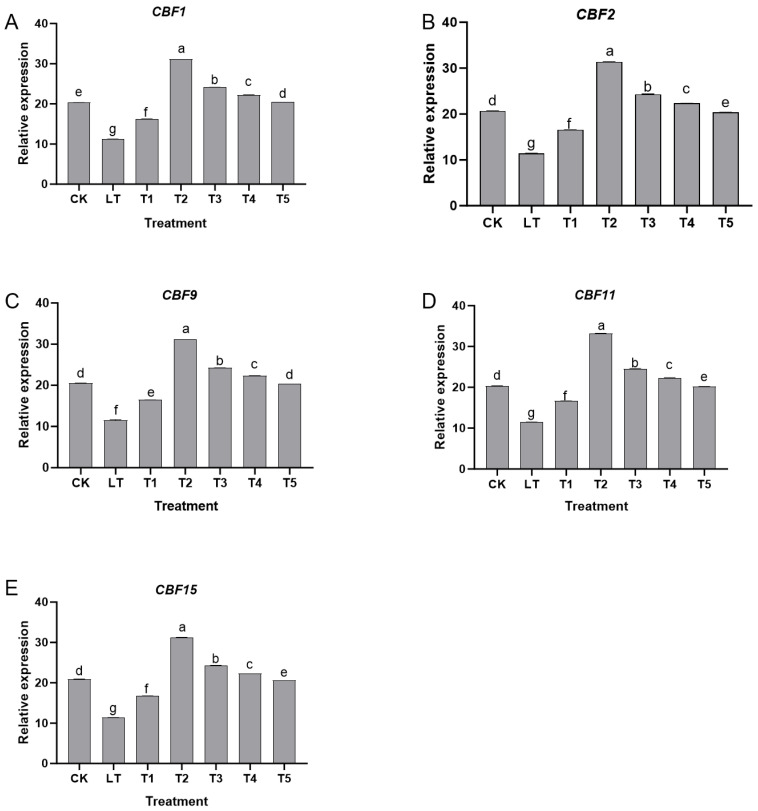
The effect of exogenous dopamine on the relative expression levels of cold-resistant genes in the *CBF* family of Shine Muscat grapes under low-temperature stress. (**A**) *CBF1* gene; (**B**) *CBF2* gene; (**C**) *CBF9* gene; (**D**) *CBF11* gene; (**E**) *CBF15* gene. Each treatment included 3 biological replicates (each replicate mixed from 3 seedlings) and 3 technical replicates for qRT-PCR. Relative expression levels were calculated using the 2^−ΔΔCt^ method with actin. Abbreviations: CK = control group (normal temperature: 25–28 °C, 1/2 Hoagland nutrient solution irrigation); LT = low-temperature stress group (0 °C, 1/2 Hoagland nutrient solution irrigation); T1–T5 = dopamine treatment groups under low-temperature stress with dopamine concentrations of 0.2, 0.4, 0.6, 0.8, and 1.0 mmol L^−1^. Different lowercase letters indicate significant differences among treatments at the *p* < 0.05 level.

In contrast, the T5 group (poor physiological tolerance) showed *CBF* expression close to the LT group (Figure 5), indicating a potential link between *CBF* gene activation and physiological cold tolerance enhancement. *CBF2* expression was positively correlated with ΦPSII (r = 0.823; *p* < 0.01), suggesting *CBF* genes may mediate Da’s regulation of photosynthesis and ion homeostasis. After treatment with exogenous dopamine, the expression levels of various genes showed regular changes with concentration: 0.2 mmol L^−1^ (T1 group) and T2 group treatments significantly upregulated *CBF* family gene expression, with the T2 group showing the most prominent effect. Compared with the LT group, the expression level of *CBF1* increased by about 2.8 times, *CBF2* increased by about 3.2 times, *CBF9* increased by about 2.5 times, *CBF11* increased by about 2.3 times, and *CBF15* increased by about 2.7 times in the T2 group, all of which were significantly higher than other treatment groups (*p* < 0.05). This indicated that appropriate concentrations of dopamine effectively activated the transcription of *CBF*-family cold-resistant genes and enhanced the plant’s cold-resistant molecular response. When the dopamine concentration was increased to 0.6 mmol L^−1^ (T3 group), the expression levels of various genes were significantly decreased compared to the T2 group (*p* < 0.05), but were still higher than the LT group. When the concentration was ≥0.8 mmol L^−1^ (T4 and T5 groups), the gene expression level further decreased. *CBF*-family gene expression level in the T5 group (1.0 mmol L^−1^) was close to or even lower than that in the LT group, indicating that high-concentration dopamine had an inhibitory effect on *CBF* gene expression.

In summary, exogenous dopamine participated in the low-temperature response of Shine Muscat grapes by regulating the expression of *CBF*-family cold-resistant genes. Treatment with 0.4 mmol L^−1^ most significantly upregulated the expression of key cold-resistant genes such as *CBF1* and *CBF2*, thereby enhancing the plant’s cold-resistant molecular mechanism. However, high concentrations of dopamine weakened the cold-resistance regulation ability of plants by inhibiting the expression of these genes. This result was consistent with the changes in physiological pathways such as photosynthetic protection and antioxidant defense, which jointly confirmed the mechanism by which exogenous dopamine synergistically enhances the cold resistance of Shine Muscat grapes through multiple pathways.

### 2.6. Correlation Analysis of Growth Indicators and Photosynthesis-, Ion-, and Oxidation-Related Indicators in Grape Plants Treated with Exogenous Dopamine Under Low-Temperature Stress

The growth status of Shine Muscat grapes under low-temperature stress (plant height, stem diameter, fresh stem weight, dry stem weight) was significantly correlated with photosynthetic system function (especially PSII stability, stomatal regulation), ion homeostasis (low Na^+^/K^+^ ratio), antioxidant defense (high SOD, CAT activity), and degree of oxidative damage (low ROS, MDA content). The correlation between antioxidant enzyme activity, ion balance, and photosynthetic parameters (Gs, Ci, Tr) and growth indicators was the strongest (|r| ≥ 0.8, *p* < 0.01). This result confirmed the comprehensive mechanism by which exogenous dopamine enhanced cold resistance through synergistic regulation of photosynthetic protection, ion homeostasis, antioxidant defense, and other pathways, providing relevant evidence for the optimal regulatory effect of 0.4 mmol L^−1^ dopamine.

## 3. Discussion

This study found for the first time that exogenous dopamine has a significant “low promotion and high inhibition” effect on the cold resistance of Shine Muscat grapes: treatment with 0.4 mmol L^−1^ can alleviate low-temperature stress to the greatest extent, while treatment with ≥0.8 mmol L^−1^ inhibits growth and weakens stress resistance. The significant positive correlation between *CBF* gene expression (upregulated 2.3–3.2 times in the T2 group) and physiological indicators (e.g., ΦPSII, r = 0.823; *p* < 0.01) suggests that dopamine may indirectly regulate *CBF*-mediated cold signaling to coordinate physiological defense pathways, though the direct molecular interaction between dopamine and *CBF* transcription factors requires further verification. Although this pattern is consistent with the role of dopamine in various other crops [7,11], this study is the first to clarify that the optimal regulatory threshold for dopamine in the Shine Muscat grape is 0.4 mmol L^−1^. Previously, Dai et al. [12] only reported the effect of dopamine on the photosynthetic performance of grapes, without involving the effect of cold-resistance concentration. The hypothesis of “concentration-dependent threshold effect of exogenous substances” proposed by Wang et al. [15] lacks grape-specific data, and this study provides key parameters for the precise application of dopamine in fruit crops, filling the research gap in this field in the grape genus. The potential mechanism may be that low concentrations of dopamine activate the antistress pathway as a signaling molecule, while high concentrations interfere with cellular redox balance or hormone signal interactions. This speculation provides direction for subsequent molecular mechanism research. This variation likely stems from differences in substance specificity: dopamine has higher biological activity in Shine Muscat and may trigger stress responses at lower concentrations.

Under low-temperature stress, the photosynthetic efficiency of Shine Muscat grapes decreased due to chlorophyll degradation, impaired PSII function, and non-stomatal limitation, which is consistent with the regulatory effect of methyl jasmonate on grapes [5]. The increase in Ci and decrease in Gs under low temperature indicates that the decrease in photosynthesis rate is not solely caused by stomatal limitation, which is a common non-stomatal limitation phenomenon in grape low-temperature stress reported in previous studies [5,17]. However, this study mainly revealed the dopamine light energy conversion process at the leaf level. Compared with the study by Wang et al. [17] in loquat, this study found that 0.4 mmol L^−1^ dopamine promoted the increase of ΦPSII and ETR, which was significantly higher than the regulatory effect reported by Luo et al. [18], indicating that dopamine has more advantages in stabilizing the structure of the photosystem and promoting light energy conversion. Low temperature induces PSII damage, and dopamine’s upregulation of *CBF* genes (not observed in non-stress studies) may specifically protect photosystem structure. In addition, Wang et al. [8] only revealed the correlation between exogenous substances and the cold resistance and photosynthetic parameters of grape branches, while this study focused on the dynamic changes in the leaf photosynthetic system, systematically analyzing the response mechanism of photosynthetic protection from dimensions such as chlorophyll synthesis, PSII functional stability, and light energy conversion efficiency, so has more advantages in terms of research specificity and depth. Dopamine synergistically regulates osmotic adjustment and ion homeostasis, two pathways previously thought to act independently under cold stress. First, it promotes proline accumulation to enhance cellular osmotic pressure, addressing dehydration stress. Second, it reduces Na^+^ influx while stabilizing K^+^ levels, maintaining ion gradient balance. This synergy ensures comprehensive cellular protection, which is more effective than single-pathway regulation reported in other grape stress studies.

In low-temperature stress, plants usually cope with stress by accumulating proline (osmotic regulation) and changing the Na^+^/K^+^ ratio (ion homeostasis) [6], but the synergistic effect of the two is not yet clear in low-temperature stress research. This study found that 0.4 mmol L^−1^ dopamine can increase proline content and decrease the Na^+^/K^+^ ratio, confirming for the first time the synergistic effect of osmoregulatory substances (proline) and ion balance under low-temperature stress. This is complementary to the ion regulation mechanism under salt stress reported by Afzal et al. [19], providing a new perspective on the relationship between plant stress resistance and metabolism. Further research reveals that the core of dopamine maintaining ion homeostasis is to inhibit Na^+^ influx rather than simply promote K^+^ absorption. This mechanism is different from the main cause of ion imbalance in grape salt stress proposed by Lu et al. [20], providing new ideas for understanding the regulation of ion transport for maintaining cellular ion gradient. In addition, Xing et al. [13] found that ion homeostasis depends on rootstock improvement, while this study confirmed that dopamine can directly regulate ion balance without relying on rootstock, providing a practical basis for simplifying cold-resistant cultivation techniques. Combined with the complexity of the ion homeostasis regulation network revealed by Liu et al. [21], the synergistic regulation results of this study further confirm that plant stress resistance is a process of multi-pathway integration. Dopamine can enhance the activity of SOD, POD, and CAT and reduce the accumulation of ROS, which is consistent with the antioxidant properties of dopamine in apples [10] and cucumbers [11]. The optimal dopamine concentration (0.4 mmol L^−1^) simultaneously enhanced antioxidant enzyme activity and stabilized ion balance. This dual regulation is critical for alleviating low-temperature-induced oxidative damage and cellular osmotic disorder, addressing two core physiological bottlenecks of Shine Muscat under cold stress. Compared with the 20–30% increase in SOD activity by dopamine in cucumbers [11], the 31.7% increase in SOD activity in the T2 group of this study indicates that dopamine has a stronger antioxidant regulatory effect in Shine Muscat grapes, which may be related to the variety-specific sensitivity of grapes to dopamine. This study confirmed for the first time through correlation analysis that the activity of SOD and CAT is significantly positively correlated with growth indicators such as plant height and stem diameter (r > 0.8, *p* < 0.01), breaking the limitation of only observing changes in enzyme activity in the past and directly proving that antioxidant capacity is the core link for dopamine to enhance cold resistance. Compared with the melatonin reported by Tang et al. [22], dopamine showed a higher increase in POD activity in the Shine Muscat grape, indicating its specific advantage in the grape enzymatic defense system. In addition, Kong [23] found that dopamine can alleviate cadmium stress in *Pyrus betulifolia* seedlings, and this study for the first time focused on the universal cross-stress response of antioxidant regulation to the low-temperature response of Shine Muscat, providing a new direction for understanding the broad-spectrum stress resistance mechanism of grapes [24].

*CBF* transcription factors are the core of plant cold-resistance pathways [16], but dopamine’s regulation of them in Shine Muscat is not yet clear. This study found that 0.4 mmol L^−1^ dopamine can increase the expression level of *CBF2* gene by 3.2 times compared to the low-temperature group, and is the first to clarify the upregulation effect of dopamine on the *CBF* family genes of Shine Muscat. This is consistent with the conservative function of *CBF* studied by Cui et al. [25], and further establishes its core position in the dopamine regulatory network. Based on the transcription factor network revealed by Lv et al. [26], this study suggests that the *CBF* family may collaborate with similar regulatory modules in grapes to enhance cold-resistance responses through multifactor interactions. This provides further research directions for understanding the molecular mechanisms of dopamine regulation.

It is necessary to acknowledge the limitations of this study. This study focused on 1-year-old seedlings under controlled artificial climate conditions. The regulatory effect of dopamine on mature grapevines in open-field environments (with fluctuating temperatures and other stresses) remains to be verified. We identified a correlation between *CBF* genes and physiological pathways, but the direct molecular targets of dopamine (e.g., whether it binds to *CBF* promoters) have not been clarified. Corresponding future research directions should include conducting field trials to validate the optimal dopamine concentration and application timing for mature Shine Muscat vines, considering regional climate differences, using molecular techniques (e.g., ChIP-seq, yeast two-hybrid) to explore the direct interaction between dopamine and CBF gene regulatory elements, clarifying the upstream signaling mechanism, investigating whether dopamine can be combined with other exogenous substances (e.g., melatonin) to achieve synergistic cold tolerance enhancement, and further improving application efficiency. These follow-up studies will help to further improve the theoretical system of dopamine regulating grape cold tolerance and provide more comprehensive technical support for cold-resistant grape cultivation, which is of great significance for promoting the stable production of cold-sensitive grape varieties in northern China.

## 4. Materials and Methods

### 4.1. Materials

The test material was 1-year-old Shine Muscat grape plants, taken from the grape germplasm resource nursery of Henan Institute of Science and Technology. Seedlings with strong growth and consistent phenotype were selected. The plants were planted in a plastic flowerpot with a diameter of 20 cm and a height of 25 cm. The cultivation substrate was a mixture of garden soil and commercial nutrient soil (purchased from Zhengzhou Green Source Horticultural Co., Ltd., Zhengzhou, China) at a volume ratio of 1:1. The basic physical and chemical properties of the substrate were pH 6.5–7.0, organic matter content 2.8–3.2%, total nitrogen 1.2 g kg^−1^, available phosphorus 65 mg kg^−1^, and available potassium 110 mg kg^−1^. Before potting, the substrate was sterilized at 121 °C for 30 min to eliminate pathogenic microorganisms and weed seeds. Before the experiment, the plants were cultivated in the greenhouse, and the environmental conditions were controlled as follows: temperature 25–28 °C, relative humidity 60–70%, light time 14–16 h d^−1^, light intensity 3000–5000 lux, and regular watering, fertilization, and pest control during the period. When a plant had grown 7–8 functional leaves and the growth was consistent, it was used for subsequent experiments.

The exogenous dopamine reagent was dopamine hydrochloride (purity ≥ 98%, Beijing Solarbio Science & Technology Co., Ltd., Beijing, China). Before use, deionized water was used to prepare the treatment solutions of 0.2, 0.4, 0.6, 0.8, and 1.0 mmol L^−1^.

### 4.2. Methods

#### 4.2.1. Experimental Design

Based on the previous research results of Wu et al. [3], Liu [10], Lan et al. [11], Wang et al. [17], Jiao [27], and Chen et al. [28], and combined with the pretest verification of this study, the experiment was designed. The selection of 0 °C as the low-temperature stress temperature is based on two factors: ① prior experiments showed that 0 °C can induce typical low-temperature stress responses (such as decreased photosynthetic efficiency, increased ROS content) in Shine Muscat grapes without causing irreversible seedling death; and ② it is consistent with the low-temperature stress temperature used in authoritative grape cold-resistance studies (e.g., Wu et al. [3] used 0 °C to evaluate the cold resistance of 129 grape germplasms), ensuring comparability of results.

The basic replication unit of the experiment was a single Shine Muscat seedling planted in a plastic pot (diameter 20 cm, height 25 cm). Each treatment group included 10 pots (10 seedlings), of which 8 seedlings with consistent growth (excluding weak or abnormal individuals) were selected as valid biological replicates for subsequent index determination. Before the experiment, all seedlings were randomly arranged in the greenhouse/artificial climate chamber using a completely random design. The positions of pots in each group were reshuffled every 2 days to avoid the influence of uneven environmental factors (e.g., light, temperature) on experimental results. The randomization process was recorded using Excel’s random number generation function to ensure traceability.

Plants with consistent growth were randomly divided into 7 groups, with 10 pots in each group (1 seedling per pot), and treated as follows: (1) Control (CK): irrigated with 1/2 Hoagland nutrient solution normally and cultivated in a greenhouse environment; (2) low-temperature stress (0 °C, low temperature (LT)): placed in an artificial climate chamber (0 °C, 12 h d^−1^ of light, 500 μmol m^−2^ s^−1^ PAR (original 3000 lux, converted using a PAR sensor, RTOP-1000Y (Top Instrument Co., Ltd., Hangzhou, China)), 70% relative humidity), irrigated with 1/2 Hoagland nutrient solution daily; (3) dopamine treatment group (T1–T5): under the same low-temperature stress conditions as the LT group, dopamine solutions of corresponding concentrations (T1: 0.2 mmol L^−1^, T2: 0.4 mmol L^−1^, T3: 0.6 mmol L^−1^, T4: 0.8 mmol L^−1^, T5: 1.0 mmol L^−1^) were irrigated every 3 days until the substrate was moist and free of standing water. The dopamine solution was prepared by mixing dopamine hydrochloride with 1/2 Hoagland nutrient solution, and each pot was irrigated with 200 mL of the mixture each time. The LT group and CK group were irrigated with 200 mL of pure 1/2 Hoagland nutrient solution at the same frequency to ensure consistent water and nutrient supply. The processing cycle was 15 days (the entire experiment duration), and the environmental fluctuations in the climate chamber were controlled within ±0.5 °C, light intensity ± 200 lux, and relative humidity ± 5% (Table 2). The site was regularly cleaned during the trial period to prevent and control pests and diseases.

#### 4.2.2. Determination of Growth Indicators

Initial plant height was recorded before treatment (from the base of the new shoot to the top growth point, accurate to 0.1 cm) to ensure no significant differences in initial growth among groups. After treatment, the following indicators were measured: plant height—measure vertically from the base of the new shoot to the top growth point with a ruler, accurate to 0.1 cm; stem diameter—measure the diameter 2 cm above the base of the new shoot using a vernier caliper with an accuracy of 0.02 mm; fresh stem weight—after washing and absorbing water from the plant, weigh the fresh weight of the stem; dry stem weight—after withering at 105 °C for 30 min, dry at 80 °C until constant weight. For growth indicators (plant height, stem diameter, etc.), 8 seedlings per group (biological replicates) were used, with 3 measurements per seedling (technical replicates). For each seedling, plant height was measured 3 times at different positions (base to top) and averaged. Stem diameter was measured 3 times at 2 cm above the base and averaged.

#### 4.2.3. Determination of Photosynthetic Parameters

Photosynthetic pigment content (Chla, Chlb, total Chl, carotenoids) was determined according to the method of Yang et al. [29]. Photosynthetic gas exchange parameters: the LI-6400XT portable photosynthesis analyzer was used to measure net photosynthesis rate (Pn), stomatal conductance (Gs), transpiration rate (Tr), water use efficiency (WUE), and intercellular CO_2_ concentration (Ci). The conditions set were atmospheric CO_2_ concentration of 380 ± 5.0 μmol mol^−1^, temperature of 25–28 °C, and relative humidity of 55–65%. Select 6 plants for each treatment and measure them after light adaptation from 8:30 to 11:30 [27]. Chlorophyll fluorescence parameters: on the 5th, 7th, and 10th days of treatment, select 5 plants and take the 3rd to 4th leaf from the growth point downwards. After 30 min of dark treatment, measure the maximum photochemical efficiency (Fv/Fm), actual photochemical efficiency (ΦPSII), electron transfer rate (ETR), and non-photochemical quenching (NPQ) using a Handy PEA portable fluorescence analyzer [27]. Each parameter was measured twice per leaf (5 biological replicates, 2 technical replicates per leaf). Five biological replicates were set because the instrument has high measurement accuracy (coefficient of variation < 5%), and five replicates can fully reflect the dynamic changes in PSII function under low-temperature stress. Increasing replicates would not significantly improve data reliability, but increase unnecessary sample consumption.

#### 4.2.4. Determination of Physiological and Biochemical Indicators

The determination of free proline (Pro) content was carried out using the ninhydrin colorimetric method [30]: take 0.1 g of fresh leaves dried at 85 °C, boil them with concentrated sulfuric acid, and treat them with H_2_O_2_ to make up to 100 mL; take 1 mL of the filtrate and measure the Na^+^ and K^+^ content using an ion chromatograph; use NaCl and KCl standard solutions to draw standard curves for quantification [19]. Ion extraction protocol: Weigh 0.1 g of dried leaf powder into a 50 mL centrifuge tube; add 5 mL of concentrated sulfuric acid (analytical grade, 98%), seal and place in a water bath at 100 °C for 3 h for digestion; after cooling, add 2 mL of 30% H_2_O_2_ (analytical grade) and continue digestion at 100 °C for 1 h until the solution is clear; transfer the digestion solution to a 100 mL volumetric flask, add ultrapure water to the marked line, and filter through a 0.22 μm filter membrane; determine ion content using an ion chromatograph with 3 parallel injections per sample. The activity of superoxide dismutase (SOD) was determined using the nitro blue tetrazole (NBT) photochemical reduction method, the activity of peroxidase (POD) was determined using the guaiacol method, and the activity of catalase (CAT) was determined by potassium permanganate titration [20,27]. The content of superoxide anions (O_2_$\overset{-}{.}$) and hydrogen peroxide (H_2_O_2_) was determined by conventional methods [30]. The content of malondialdehyde (MDA) was determined using the thiobarbituric acid colorimetric method. The relative conductivity (REC) was measured using a DDSJ-308F conductivity meter.

#### 4.2.5. Determination of Gene Expression Level

Target genes: select *CBF* family cold-resistant genes (*CBF1*, *CBF2*, *CBF9*, *CBF11*, *CBF15*) [16]. RNA extraction and reverse transcription: take 0.1 g of leaves and extract total RNA using the Tiangen DP430 RNA extraction kit. Measure the concentration and purity using NanoDrop2000 (Thermo Scientific, Waltham, MA, USA) (A_260_/A_28_ = 1.8–2.0); First-strand cDNA was reversely transcribed using the FastKing cDNA First Strand Synthesis Kit (Genomic DNA-free) (KR116) (Tiangen Biotech Co., Ltd., Beijing, China). qRT-PCR: use the BioNTech RR420A (Takara Bio Inc., Shiga, Japan) SYBR Green kit with reaction system of 20 μL (containing 10 μL SYBR Green Mix, 0.8 μL upstream and downstream primers (10 μmol L^−1^), 2 μL cDNA, and 6.4 μL ddH_2_O). Reaction conditions: pre-denaturation at 95 °C for 30 s, denaturation at 95 °C for 5 s, annealing at 60 °C for 30 s, 40 cycles. Using the actin gene as an internal reference, the relative expression level was calculated using the 2^−ΔΔCt^ method.

#### 4.2.6. Statistical Analysis

Data were organized and managed using Microsoft Excel 2010. All results are presented as means ± standard deviation (SD). Statistical analyses were performed using SPSS 26.0. One-way analysis of variance (ANOVA) was applied to examine significant differences among groups. The significance level was set at *p* < 0.05. Significant differences detected were followed by Duncan’s new multiple-range test for post hoc multiple comparisons. Pearson correlation analyses were conducted via SPSS 26.0 to calculate correlation coefficients between different indicators. All statistical graphs were generated and optimized using GraphPad Prism 10.1.2 software.

## 5. Conclusions

This study found that exogenous dopamine can enhance the cold resistance of Shine Muscat grapes through a multi-pathway synergistic network that coordinately regulates key physiological processes such as photosynthetic protection, osmotic adjustment, ion homeostasis, antioxidant defense, and cold-responsive gene expression. Furthermore, the study revealed the correlation mechanism between different physiological processes and gene expression, among which the antioxidant defense pathway is the core pathway for dopamine-mediated growth recovery of grapes under low-temperature stress. The results of this study not only help to clarify the cold-resistance regulation mechanism of Shine Muscat grapes under low-temperature stress and provide reference parameters for the application of exogenous regulatory substances, but also clearly identify 0.4 mmol L^−1^ as the optimal concentration for exogenous dopamine regulation. This finding fills the data gap in the research on dopamine in the cold resistance of Shine Muscat grapes, provides theoretical support for the scientific application of dopamine in cold-resistant grape cultivation, and also offers research ideas and a practical basis for stress-resistance improvements to similar cold-sensitive fruit tree varieties. It should be noted that this study was only conducted on Shine Muscat grape seedlings under specific conditions. Future research can further explore the impact of different environmental factors on the regulatory effect of dopamine and verify the applicability of relevant mechanisms in other grape varieties. At the same time, field experiments can be carried out to clarify the cold-resistance regulatory effect of dopamine on mature grape plants, thereby promoting its practical application in cold-resistant grape cultivation and production.

## Figures and Tables

**Table 1:** Correlation analysis of growth indicators of grape plants treated with exogenous dopamine (Da) under low-temperature stress and related indicators of photosynthesis, ions, and oxidation (r values).

	Plant Height	Stem Diameter	Fresh Stem Weight	Dry Stem Weight
Chla	0.1077	0.09187	−0.26078	−0.32093
Chlb	0.12135	0.06093	−0.26184	−0.32147
Chla + b	−0.64891	−0.39574	−0.85662	−0.88894
Car	0.73695	0.52323	0.65446	0.6324
PN	0.43352	0.65015	0.78333 *	0.81822 *
GS	0.58507	0.78355 *	0.87909 **	0.90935 **
TR	0.6012	0.86806 *	0.88209 *	0.89183 *
WUE	0.73933 *	0.97288 **	0.81611 *	0.80555 *
CI	0.67827	0.95469 **	0.86907 **	0.86475 **
d5 Fv/Fm	0.45991	0.80184 *	0.83018 *	0.85992 *
d7 Fv/Fm	0.70093	0.7779	0.89469 *	0.89287 *
d10 Fv/Fm	0.14116	0.35434	0.42719	0.43947
Pro	−0.12694	−0.14892	−0.50965	−0.56386
Na+	−0.68159	−0.85343 *	−0.91756 **	−0.93449 **
K+	−0.36143	−0.58398	−0.7302 *	−0.78544 *
Na+/K+	−0.71254	−0.88608 **	−0.92955 **	−0.93887 **
SOD	0.85445 **	0.92459 **	0.97943 **	0.9794 **
POD	0.62956	0.7298 *	0.86236 **	0.88695 **
CAT	0.77384 *	0.89169 **	0.95583 **	0.95881 **
O_2_$\overset{-}{.}$	−0.72521 *	−0.98337 **	−0.86899 **	−0.86683 **
H_2_O_2_	−0.56209	−0.90176 **	−0.74678 *	−0.74055 *
MDA	−0.67538	−0.94056 **	−0.74654 *	−0.72921 *
REC	−0.6469	−0.88402 **	−0.88407 **	−0.90381 **

Note: when |r| ≥ 0.8, it indicates a strong correlation; when 0.5 ≤ |r| < 0.8, it is a strong correlation; when 0.3 ≤ |r| < 0.5, the correlation is moderate; when |r| < 0.3, the correlation is weak. * Significant correlation at the *p* < 0.05 level; ** highly significant correlation at the *p* < 0.01 level.

**Table 2:** Experimental design of exogenous dopamine regulating Shine Muscat grape under low-temperature stress.

Group Name	Treatment Conditions	Environmental Parameters	Dopamine Application	Irrigation Substance	Application Frequency
CK (control)	Normal growth	Greenhouse: 25–28 °C, 60–70% RH, 14–16 h light (400 μmol m^−2^ s^−1^ PAR)	None	1/2 Hoagland nutrient solution	Daily
LT (low-temperature stress)	0 °C stress	Artificial climate chamber: 0 °C, 70% RH, 12 h light (500 μmol m^−2^ s^−1^ PAR)	None	1/2 Hoagland nutrient solution	Daily
T1 (Da treatment)	0 °C stress + Da	Same as LT group	0.2 mmol L^−1^ dopamine	Da solution (mixed with 1/2 Hoagland)	Every 3 days
T2 (Da treatment)	0 °C stress + Da	Same as LT group	0.4 mmol L^−1^ dopamine	Da solution (mixed with 1/2 Hoagland)	Every 3 days
T3 (Da treatment)	0 °C stress + Da	Same as LT group	0.6 mmol L^−1^ dopamine	Da solution (mixed with 1/2 Hoagland)	Every 3 days
T4 (Da treatment)	0 °C stress + Da	Same as LT group	0.8 mmol L^−1^ dopamine	Da solution (mixed with 1/2 Hoagland)	Every 3 days
T5 (Da treatment)	0 °C stress + Da	Same as LT group	1.0 mmol L^−1^ dopamine	Da solution (mixed with 1/2 Hoagland)	Every 3 days

Note: RH = relative humidity; PAR = photosynthetically active radiation. Each group had 10 pots (1 seedling per pot) as the basic experimental unit.

## Data Availability

All data generated or analyzed during this study are included in this published article.

## Abbreviations

Da: dopamine; Chla: chlorophyll a; Chlb: chlorophyll b; Car: carotenoid; Pn: net photosynthesis rate; Gs: stomatal conductance; Tr: transpiration rate; WUE: water use efficiency; Ci: intercellular CO_2_ concentration; PSII: photosystem II; Fv/Fm: maximum photochemical efficiency; ΦPSII: actual photochemical efficiency; ETR: electron transfer rate; NPQ: non-photochemical quenching; Pro: proline; Na+: sodium ion; K+: potassium ion; SOD: superoxide dismutase; POD: peroxidase; CAT: catalase; O_2_$\overset{-}{.}$: superoxide anion; H_2_O_2_: hydrogen peroxide; MDA: malondialdehyde; REC: relative conductivity; CBF: C-repeat binding factor; qRT-PCR: quantitative real-time polymerase chain reaction.
